# Transparency and conflicts of interest disclosure at an international periodontology conference

**DOI:** 10.1111/eos.70075

**Published:** 2026-03-12

**Authors:** Clovis Mariano Faggion

**Affiliations:** ^1^ Department of Periodontology and Operative Dentistry University Hospital Münster Münster Germany

**Keywords:** bias, biomedical ethics, conferences, conflicts of interest, ethics

## Abstract

The objectives of this study were threefold: (1) to evaluate whether presenter information related to potential conflicts of interest (COIs) is adequately disclosed on the official EuroPerio and European Federation of Periodontology (EFP) websites; (2) to assess how speakers report COI‐related information during their presentations; and (3) to examine the criteria used for selecting conference presenters. The programme and video presentations from EuroPerio 11 (May 2025) were systematically analysed. Among 163 invited presenters, only six (4%) disclosed potential COIs on the conference website, and these disclosures lacked detail. Of 160 analysed presentations, 30.1% declared no COI, 19.6% declared potential COIs, and 48.5% made no disclosure; the median disclosure duration was seven seconds. Observations revealed that COI statements were typically brief, suggesting that disclosure was often treated as a formality rather than a substantive ethical practice. No explicit or publicly accessible criteria for presenter selection were identified on either the conference or federation websites. These findings indicate substantial underreporting of both financial and non‐financial COIs, limited enforcement of disclosure practices, and a lack of transparency in presenter selection. Clear, standardised, and verifiable procedures for COI reporting and presenter selection are needed to strengthen scientific integrity and maintain audience trust.

## INTRODUCTION

Scientific dental conferences play a crucial role in disseminating information to clinicians [1], thereby enhancing their decision‐making and ultimately improving patient outcomes. These meetings employ various formats, including lectures, workshops and poster presentations, to share knowledge. To uphold ethical standards, it is essential that the information presented remains as unbiased as possible.

Attendees must be fully informed not only about the lecture content but also about the professional background of the presenter. Potential conflicts of interest (COI) may influence how the audience perceives and interprets the presented evidence. For instance, a presenter discussing the effectiveness of a specific drug may selectively report findings [2] that favour its efficacy. If the presenter has affiliations with the company that manufactures the drug, this relationship could impact the credibility of the findings. Consequently, scientific conference organizers must ensure transparency by disclosing all relevant information regarding the presenters' potential COI, enabling attendees to critically assess the presented evidence.

The issue of COI among authors and editorial board members is extensively reported and discussed in the scientific literature [3, 4, 5, 6, 7, 8, 9, 10, 11, 12, 13]. Regarding the reporting of COI declarations in articles published in dental journals, existing evidence is conflicting. Some studies [5, 6] suggest underreporting of COI, whereas others [7] report high levels of disclosure across various study types.

The reporting of COI in medical conferences has also been comprehensively addressed in the literature [14, 15, 16, 17, 18, 19, 20, 21]. In contrast, evidence regarding COI declarations by speakers at dental conferences remains limited. For instance, a study [22] assessing disclosures at an oral and maxillofacial surgery dental implant conference, concluded that most speakers underreported payments received from companies with interests relevant to the conference topic.

EuroPerio is the largest international meeting dedicated to periodontology, organized by the European Federation of Periodontology (EFP) [23]. This triennial event attracts leading experts in the field along with thousands of clinicians who seek to integrate new knowledge into their clinical practice. Given the significance of these conferences in shaping clinical decision‐making, it is imperative that attendees are provided with adequate information to accurately interpret the evidence presented.

Medical conference presentations sometimes lack transparent and meaningful COI disclosure, which may affect how audiences interpret speaker content. In one prospective observational study of oral presentations at multiple medical conferences [15], 29% did not include any COI statement at all, and many disclosures were displayed so briefly that delegates could not reasonably read them. In cases where COIs were disclosed, presenters rarely discussed their relevance to the content being presented, and discordances existed between spoken disclosures and written formats. This suggests that current COI practices at conferences may be insufficient for assessing potential bias in speaker content.

Additional observational research from national trauma meetings [24] found that patterns of COI disclosures vary widely by role (e.g., speaker, moderator) and that many participants with documented financial relationships did not disclose them, highlighting inconsistent implementation of COI policies across professional meetings. The authors concluded that explicit, standardized COI disclosure policies could improve reporting and help preserve an evidence‑based environment free from commercial influence.

Overall, while COI disclosure policies exist for research publications, the available evidence shows that conference COI reporting practices are often non‑transparent and inconsistently applied, thereby limiting a delegate's ability to assess potential influences on speaker objectivity [15].

Another ethical consideration potentially related to COI concerns the criteria used by scientific conferences to select presenters. In the interest of transparency and research integrity, these criteria should be publicly accessible. However, it remains unclear whether they are currently available to the general public.

The objectives of this study were threefold: (1) to evaluate whether presenter information related to potential COIs is adequately disclosed on the official EuroPerio and EFP websites; (2) to assess how speakers report information in their presentations that could indicate potential COIs; and (3) to examine the criteria used for selecting conference presenters.

## METHODS

### Definition of COI

In the context of the present research, the definition of COI proposed by Cochrane was adopted: “A conflict of interest is defined as a set of conditions that pose a risk that professional judgement concerning a primary interest (such as patients' welfare or the validity of research) can be unduly influenced (consciously or unconsciously) by a secondary interest (such as financial gain).” [25]. For the purposes of this study, the concept of secondary interests was further extended to include not only financial but also non‐financial gains.

### Research Questions

This study was guided by the following research questions:

- Is information potentially indicating a financial COI adequately reported in the official program published on the EuroPerio website?
- Do presenters disclose their potential COIs during their conference presentations?
- Does the EuroPerio website provide clear criteria for the selection of presenters at the conference?

### Eligibility Criteria and Rationale for Selecting this Meeting

This meeting was selected because it is one of the most well‐organized, reputable and largest events in the field of dentistry, attracting thousands of researchers, clinicians and students from around the world. Additionally, the conference maintains strong connections with the dental industry, making it a particularly appropriate platform for evaluating COIs among participants. Consequently, it is hypothesized that other conferences would likely demonstrate similar or less rigorous practices with respect to COI reporting.

This case study focused on the EuroPerio 11 Meeting, held from 14 May to 17 May 2025. The analysis was limited to the EuroPerio 11 and EFP websites, excluding other EFP conferences, as EuroPerio 11 represents the most recent benchmark in transparency and scientific disclosure.

### Data Search, Selection and Extraction

Initially, the program section of the EuroPerio 11 website was systematically reviewed for presentations scheduled between 14 May and 17 May 2025. The programme was assessed via the following link: https://europerio11.abstractserver.com/program/#/program/2/horizontal. Each presentation slot was examined for presenter‐related information. Relevant data were extracted and recorded in an Excel file (Supporting Information) for subsequent analysis, including: (1) presenter's country; (2) type of presentation (lecture or research session); (3) date of the lecture/research session; (4) lecture support (industry‐sponsored or non‐industry‐sponsored); (5) focus of the lecture/research session (therapeutic interventions or non‐interventional topics); and (6) any reported information that could indicate potential COI of the presenters.

To address Research Question 1, I analysed the proportion of presenters who adequately disclosed potential COIs in the official program. Specifically, the analysis evaluated whether presenters reported any industry affiliations or explicitly stated the absence of COIs.

To address Question 2, presentation videos were analysed to determine whether presenters disclosed potential COIs and to evaluate the quality of these disclosures. The assessment included both the full talk of each presenter, focusing on COI‐related information, and an evaluation of the corresponding presentation slide. The analysis considered the following criteria: (1) whether the presenter explicitly stated the absence of a COI; (2) whether a COI was declared; (3) whether no statement regarding COI was made; (4) the duration of the disclosure; and (5) whether the standard COI form provided by the conference organizers was used (see Supporting Information).

The slide assessment was conducted as follows: if the presenter stated on the slide that there was no COI, the slide was scored as 1 (NO COI), even when the statement appeared paradoxical—for example, when the speaker declared no COI but the slide contained information that could suggest a potential COI. If no slide was presented but the presenter verbally stated that there was no COI (without providing further information), this was likewise scored as 1 (NO COI). The variable time referred to the duration for which the COI slide was visible to the audience. In cases where no slide was presented, time corresponded to the duration of the verbal disclosure. Additional methodological details related to Question 2 are provided in Supporting Information.

To address Research Question 3, information was collected from both the conference and Federation websites. These sources were examined to identify any explicit criteria or patterns in the selection of presenters, such as institutional affiliation, prior collaborations, or professional networks. This approach aimed to explore potential influences on presenter selection beyond formal financial sponsorships, such as professional relationships or organizational preferences, which could indicate non‐financial conflicts of interest. Where available, this information is reported in the current article.

### Ethics and Data Protection

This study analysed publicly presented material from EuroPerio 11 video recordings, which were accessible exclusively to registered conference participants. The author did not attend the meeting in person but reviewed the video recordings together with a colleague who participated in the conference and declined both authorship and identification.

All data are reported in aggregated form, without inclusion of presenter names or other personally identifiable information. Although presenter statements were transcribed for analytical purposes, no identifiable data are included in the manuscript or supplementary materials. In accordance with the General Data Protection Regulation (GDPR), original video transcripts and any potentially identifiable information cannot be shared.

As the study involved no interaction with human participants and analysed existing conference materials, formal ethical approval or review by an Institutional Review Board was not required.

## RESULTS

### Lecture Characteristics

More than 10,000 participants from 107 countries attended the conference [26], which featured 74 main lectures and 19 research sessions comprising 150 short presentations. Main lectures typically lasted 90 min and included about three speakers. Research sessions generally ran for 100 min and featured seven to eight speakers. Of the main lectures, 40.5% (30 lectures) were sponsored by the dental industry. In terms of content, 73 main lectures and research sessions focused on therapeutic interventions, while 19 addressed non‐interventional topics. The event also hosted 112 exhibitors and 26 sponsors.

### Reporting of COI Statements on the Congress Website (Research Question 1)

Out of 163 invited presenters ('Invited Faculty—European Federation of Periodontology', n.d.), only six (4%) disclosed potential financial COIs, although these disclosures lacked specific or detailed information. Examples of disclosures included:

• The speaker has obtained multiple research grants from institutional and private sources to advance their scholarly work.
• The speaker's research activities have been supported through collaborations and funding from industry partners.
• The speaker has accumulated extensive professional experience across diverse sectors of the industry.

### Reporting of COI Statements During Presentations (Research Question 2)

Forty‐two video presentations were available from the EuroPerio website, comprising a total of 160 speakers. Among these presenters, 49 (30.1%) explicitly declared no COIs, 32 (19.6%) declared potential COIs, and 79 (48.5%) did not provide any statement regarding potential COIs. For two presenters (1.2%), the disclosure status could not be determined.

Presenters spent a median of 7 s (interquartile range [IQR], 4–15 s) reporting their COI statements. The mean duration for presenting COI statements was 9.1 s (SD 10.2). Three presenters (1.8%) delivered their statements in less than 1 s.

Among those who reported a COI statement, 39 (23.9%) used the standardized disclosure form provided by the congress organizers, whereas 38 (23.3%) used a free‐text format. In four cases (2.5%), it was not possible to determine from the video whether a COI disclosure slide was shown.

### Criteria for Selecting Presenters (Research Question 3)

No clear or specific information regarding the criteria used to select presenters was found on either the conference or federation websites, although the EuroPerio Procedures document [27] outlines general procedures for presenter selection (see Table 1). The document suggests a preference for European speakers when candidates are deemed to have equal competence; however, the qualifications or criteria used to assess such competence are not specified.

**TABLE 1:** Procedures used by the Scientific Chairman and the organizing committee to select presenters in Europerio conferences.

**Scientific Program Development**: The Scientific Chairman, in collaboration with the Organizing Committee, is responsible for creating the scientific program, which includes inviting speakers. National Societies of the EFP are invited to propose possible speakers and topics to assist in finalizing the program. **Speaker Selection**: The Organizing Committee, on the proposal of the Scientific Chairman, selects the speakers for each session. For equal competencies, European speakers are preferred. However, speakers must not be contacted until the scientific program is presented to the General Assembly. **Abstract Selection**: A call for abstracts is made 1 year before the Congress. Abstracts are submitted via an electronic system and are reviewed by the Abstract Selection Committee. This committee is responsible for reviewing and selecting abstracts for presentation at the Congress. They also determine the mode of presentation (oral, poster, etc.). **Industry Sessions**: Industry partners propose subjects, speakers and chairmen for the Industry sessions, which are subject to approval by the Organizing Committee. The Congress Committee oversees the scientific integrity of these sessions and can intervene in case of conflict. **Exemptions and Benefits for Speakers**: Invited Session Chairs and Speakers are exempted from registration fees and are compensated for travel and accommodation costs according to the rules set by the Organizing Committee. However, they do not receive an honorarium. EuroPerio speakers should refrain from presenting or speaking at other EFP National Society meetings 3 months before or after EuroPerio.

## DISCUSSION

### Main Findings

The analysis revealed a significant underreporting of both financial and non‐financial COIs among presenters at the EuroPerio 2025 meeting. The majority of presenters failed to disclose any potential COIs during their presentations. In addition, neither the conference nor the federation websites provided transparent criteria for the selection of presenters.

### Interpretation of the Results

For authors submitting a manuscript for publication, four categories of information have been identified as requiring disclosure [28]: (1) associations with commercial enterprises that provided support for the work reported in the manuscript; (2) associations with commercial entities that could reasonably be perceived as having an interest in the general subject matter of the manuscript; (3) comparable financial associations involving a spouse or children under the age of 18; and (4) non‐financial associations that may be relevant to the submitted work. These criteria could be applicable to speakers at scientific conferences, as the primary purpose of both a manuscript and a presentation is to inform an audience. A failure to disclose COI may occur not only when authors omit information that could give rise to a COI, but also when no COI exists and the authors neglect to explicitly state this.

During their presentations, the vast majority of speakers did not disclose any information regarding potential COIs, and those who did typically addressed the issue only briefly—often for just a few seconds. The present study observed a slightly longer display time for COI slides compared to other medical specialties [15, 29]; however, a median duration of 7 s may remain insufficient for the audience to adequately read and interpret the information presented, especially as some presenters reported connections with more than ten companies. This pattern may reflect a perceived lack of importance attributed to COI disclosure by the presenters themselves. It is also plausible that some presenters are reluctant to reveal industry affiliations, fearing such disclosures might undermine the perceived credibility of their findings. While COI declarations are often included verbally or on an introductory slide, prior evidence indicates that they are typically brief and shown only momentarily [15], limiting the audience's ability to absorb the information.

It is important to note that each presentation session included a moderator, who was also considered a presenter for the purposes of this study. Many moderators contributed by presenting data at the beginning of the session or by actively engaging in discussions to stimulate further dialogue. Given their influential role, it is reasonable to assume that the information provided by moderators may have an impact comparable to that of the main presenters. Therefore, the disclosure of potential COIs by moderators is as important as that of the presenters.

Several hypotheses may explain the lack or inadequate reporting of potential COIs.

First, academic and conference cultures often downplay COI disclosures, treating them as formalities rather than substantive ethical safeguards. This perspective is supported by both literature and observational evidence. Critics contend that such declarations can be burdensome and may unfairly stigmatise researchers with industry ties, implicitly portraying them as ethically compromised without clear evidence. This practice has been compared to a form of academic ‘McCarthyism,’ where disclosure requirements generate unwarranted suspicion regarding scientists’ integrity [30].

Second, presenters and organisers may fear that disclosing ties with for‐profit companies could reduce the perceived credibility of the presented findings or damage professional reputation. For example, a presenter discussing promising results related to a new product might withhold such information to avoid casting doubt on their objectivity. This concern is compounded when selective reporting—whether conscious or unconscious—emphasizes favourable outcomes, introducing bias or resembling forms of publication bias [31].

Third, omitting any reference to sponsorship or COIs may offer presenters greater flexibility in avoiding scrutiny. While a declaration is definitive and subject to accountability, non‐disclosure allows for deflection, often by attributing the omission to external factors such as the organising committee. Common justifications might include: ‘I wasn't asked to disclose anything’, or ‘It was the responsibility of the organizers’. Indeed, the current format of presenter biographies on the EuroPerio 11 platform may support this form of deflection. It appears that the responsibility for content was delegated entirely to presenters. One biography even contained internal instructions from the organising committee, indicating a lack of editorial oversight. These biographies primarily highlight achievements, awards and professional accolades, yet fail to provide specific or detailed information regarding financial or non‐financial COIs.

Fourth, speakers may choose not to disclose, or may misrepresent, COI due to motivated reasoning or concerns about their reputation. People engage in motivated reasoning [32] when processing information that threatens their self‐concept or interests. For example, a speaker might convince themselves that their judgement is unaffected and therefore see no need to disclose funding sources. To the individual, this omission may feel ethically justified, even though it ultimately undermines transparency.

Fifth, fear of losing funding, partnerships, or institutional prestige discourages full disclosure. Researchers and institutions may have incentives to underreport conflicts, particularly when financial interests are at stake [33]. Disclosure decisions may therefore be influenced by concerns about maintaining relationships with sponsors or preserving institutional reputation.

Sixth, speakers may omit or misrepresent COIs due to unclear definitions or misunderstandings about what should be disclosed. A study conducted by Mecca et al. (2015) [27] involving 64 university researchers revealed that many lacked a clear understanding of whether specific types of information—such as financial ties, institutional affiliations, or personal involvement—could influence the outcomes of their research. This ambiguity extends into public academic forums, where disclosure practices appear inconsistent and sometimes superficial.

Seventh, minimal enforcement or accountability for failing to disclose COIs reduces the incentive to comply. At EuroPerio 11, for instance, while some speakers followed the recommended procedures and used standardized forms, many others provided COI information informally or inconsistently. The absence of enforcement measures or follow‐up evaluations allows such variability in disclosure practices, potentially undermining the transparency and credibility. When there is no accountability for incomplete or inaccurate reporting, disclosure obligations may be perceived as merely symbolic rather than substantive.

In dentistry, there is a scarcity of research examining COI reporting at conferences. One study [22] investigated this issue by comparing speakers’ disclosures on the website of the American Association of Oral and Maxillofacial Surgery Dental Implant Conference 2018 with the payments reported in the Centers for Medicare and Medicaid Services Open Payments Database. Using the number of companies disclosed by speakers as the predictor variable, the study found that 35 of the 43 assessed speakers (81.4%) had received payments related to dental implants recorded in the Open Payments Database, yet this information was not disclosed on the conference website. These findings are consistent with the direction of the present research, suggesting underreporting of COI in conference settings.

With regard to the criteria for speaker selection, although the EFP website provides general guidance on conference organization (see Table 1), these documents do not specify explicit criteria for the selection of presenters. Nationality—specifically European—was noted as a consideration when presenter skills were deemed comparable, although the definition of ‘skills’ remains unclear [27]. The guidelines primarily address logistical arrangements, such as covering travel and accommodation expenses for keynote speakers, whereas other presenters are expected to fund their own participation.

### Why is detailed COI reporting important?

The medical and dental conference landscape is characterized by significant involvement from industry stakeholders. This was evident at EuroPerio 11, where 40% of the sessions were sponsored by dental industry partners. Transparency regarding any relationship between presenters and sponsors is therefore essential for maintaining the integrity of scientific discourse. Moreover, a Cochrane systematic review [34] has shown that trials funded by the manufacturer of the product being tested are more likely to yield favourable results, underscoring the need for full transparency in all scientific communications.

Another important category of potential COIs involves non‐financial interests [35], which are often more difficult to identify and disclose due to their inherently subjective nature. Nonetheless, these interests can significantly compromise the objectivity of scientific presentations. A prominent example arises in the selection of speakers for scientific meetings and conferences, where transparency in the selection process is essential. Key questions include whether presenters were chosen based on their research contributions and publication record in the relevant field, or whether they were deemed the most qualified individuals to deliver specific content—and by what criteria. Alternatively, were selections influenced by personal relationships with members of the organizing committee? In the absence of clearly defined and publicly accessible criteria, such concerns are inevitable. A lack of transparency may foster perceptions that personal affiliations, such as friendships, played a role in selection decisions.

Some may argue that inviting friends to present at high‐profile events mirrors the practice of involving personal acquaintances in the development of clinical guidelines—a process that, according to Ioannidis (2018) [36], can reinforce ‘recognizable and sustainable hierarchies of clan power’. Although this comparison is speculative, it highlights a broader concern: social relationships can shape who gains access to influential scientific roles, whether as conference speakers or guideline panel members. In both contexts, opportunities may circulate within small, well‐connected groups. Regardless of the setting, personal relationships must not be allowed to compromise scientific integrity.

### Recommendations for improvement

A critical component of COI disclosure is ensuring that presenters are not solely responsible for determining what constitutes a potential COI. Presenters should be required to disclose all relevant relationships or affiliations with individuals, organizations, or companies that could be perceived as COIs. The responsibility for evaluating the relevance of these disclosures should lie with the audience or readers, not with the presenters themselves. Without full transparency in the disclosure process, an objective and comprehensive assessment of the presented information becomes difficult—if not impossible.

One could argue that the duration of a presentation may limit the extent to which potential COI are reported. This barrier could be avoided if information on potential COI is provided in advance, along with the speaker's biography, in the scientific programme. Any updates or changes to the COI could then be briefly explained by the speaker before their presentation.

Regarding the selection of speakers at scientific meetings, measures could include invitations being made by an independent committee appointed by the meeting's organizers, which would develop predefined and objective criteria for speaker selection. The entire process—from establishing the criteria (for example, based on research performance) to issuing the invitations—could then be made publicly available. One can also consider that gender balance among speakers should be taken into account when inviting participants to scientific conferences. Analyses of academic meeting programs indicate that women are often under‑represented among invited speakers, and many panels are composed entirely of men [37].

Given that COIs may exert both conscious and unconscious influence on those presenting information, mitigation efforts should also target the audience—namely, those receiving the information. A foundational approach involves integrating education on ethical principles, including COI, into the dental curriculum. Dental students and early‐career professionals should receive formal training on how COIs can affect research outcomes and their interpretation. By fostering this ethical awareness, future professionals will be better equipped to critically appraise scientific evidence presented at conferences.

Although empirical evidence suggests that disclosure of conflicts of interest does not necessarily reduce bias and may have limited or unintended effects on decision‐making, transparency remains a foundational ethical principle in scientific research. Evidence from UK healthcare shows that disclosure systems are often incomplete, difficult to access, and poorly understood, with high prevalence of undisclosed financial conflicts and limited ability among the public to locate or interpret declarations.[38] The declaration of COI should therefore not be understood solely as an instrumental mechanism to correct bias, but as a necessary condition for research integrity, accountability and respect for readers’ autonomy. While transparency alone may be insufficient to mitigate bias, the absence of transparency undermines trust and compromises the ethical foundations of scientific practice.

### Strengths and limitations

This study is among the first to examine in detail how potential COIs are reported by presenters at dental scientific conferences, analysing both the official conference programme and video recordings of presentations. Limitations include incomplete access to recordings, which may have excluded sessions with industry support where disclosures could differ, and the focus on specific disciplines—periodontology and implant dentistry. Nevertheless, EuroPerio is a highly regarded conference and may reasonably reflect best‐practice standards in the field, suggesting that COI disclosure at other dental conferences could be less rigorous.

In reporting financial COI significant limitations in the source data were identified. Many presenters verbally reported no COIs, yet their slides revealed grants, honoraria, or participation in industry‐sponsored activities. Non‐financial COI, such as advisory roles, board memberships, or institutional leadership positions, were rarely specified or appeared briefly in slides, making them difficult to capture. Given this lack of systematic reporting, it was not possible to reliably distinguish non‐financial from financial COIs. Accordingly, the analysis focuses primarily on disclosed COIs, without assuming unreported non‐financial interests. This limitation may lead to an underestimation of the overall prevalence of COIs, which is transparently acknowledged. Another potential limitation is the inability to determine or verify whether the disclosures provided by the speakers are complete or accurate. Finally, although some presentations were recorded on video, logistical constraints prevent their public release, thereby limiting reproducibility and opportunities for further research.

## CONCLUSIONS

To uphold transparency and preserve the credibility of scientific content, conference organizers should require detailed and standardized disclosures of both financial and non‐financial COIs. Such disclosures should include clear and publicly accessible information about presenters’ professional roles and industry affiliations. Establishing a centralized and verifiable disclosure system, such as a standardized registry for conference presenters, modelled on systems such as Open Payments or tailored specifically for scientific meetings, would substantially enhance audience trust and scientific integrity.

Furthermore, the criteria used to select speakers should be explicitly defined and made publicly available. Given that the influence of major scientific conferences extends beyond the professional community into the public domain, their organizational and ethical procedures should meet the same high standards of transparency and accountability expected of public‐facing scientific institutions.

## AUTHOR CONTRIBUTIONS

**Conceptualization**: Clovis Mariano Faggion Jr. **Methodology**: Clovis Mariano Faggion Jr. **Formal analysis**: Clovis Mariano Faggion Jr. **Investigation**: Clovis Mariano Faggion Jr. **Data curation**: Clovis Mariano Faggion Jr. **Writing – original draft**: Clovis Mariano Faggion Jr. **Writing – review & editing**: Clovis Mariano Faggion Jr. **Visualization**: Clovis Mariano Faggion Jr. **Supervision**: Clovis Mariano Faggion Jr. **Project administration**: Clovis Mariano Faggion Jr.

## CONFLICT OF INTEREST STATEMENT

The author declares no conflicts of interest.

## FUNDING INFORMATION

The author has nothing to report.

## Supporting information



Supporting Information

Supporting Information

## Data Availability

The data analysed in this study were extracted from publicly presented conference materials (programme information and recorded presentations accessible to registered participants for a limited period). All relevant aggregated data and methodological details are reported within the article and its supplementary material. Due to access restrictions to the original conference recordings and data protection considerations, the underlying extracted dataset and presentation transcripts cannot be made publicly available.
