# Supplementary material for: Transparency and conflicts of interest disclosure at an international periodontology conference
Source: Eur J Oral Sci. 2026 Mar 12;134(3):e70075. doi: 10.1111/eos.70075 (PMC13181539; doi:10.1111/eos.70075)
Supplement: Supplementary file 1 — Supporting Information [file EOS-134-e70075-s002.docx]

**Supplementary file**

**Methodological Approach to Assessing Conflict of Interest Disclosures in Conference Presentations**

This study was designed to address the following research questions:

1. Is information potentially indicating a financial COI adequately reported in the official programme published on the EuroPerio website?
2. Do presenters disclose their potential COIs during their conference presentations?
3. Does the EuroPerio website provide clear criteria for the selection of presenters at the conference?

In addressing Research Question 2, several methodological challenges emerged. Initially, the researcher intended to contact the conference organisers to obtain recordings of the presentations for analytical purposes. Anticipating a potential refusal, the researcher instead sought assistance from a colleague who had attended the conference to gain access to the recordings. Attendees were permitted to access the video recordings for a limited period. Together, the researcher and the colleague reviewed the material to evaluate the disclosure of potential COIs, thereby fulfilling the primary objective of the investigation. For personal reasons, the colleague declined formal participation in the study and requested anonymity.

A central consideration in the methodology is the balance between research transparency and the privacy of presenters. While including identifiable names in analyses could provide a more detailed understanding of COI disclosure practices, this approach raises ethical concerns regarding the publication of personal and professional information. Conversely, anonymizing data protects individual privacy but may obscure patterns of non-disclosure, potentially limiting the transparency and reproducibility of the research. In this study, data are presented in aggregated form to protect privacy, while the complete tabulated dataset has been retained securely by the researcher to ensure reproducibility and allow for future scholarly scrutiny.

Subsequently, the researcher contacted the conference organisers directly to request access to the complete presentation recordings. This request was denied, even when the purpose of the inquiry was explicitly identified as academic research, confirming the anticipated access limitations.

This methodological reflection highlights a broader discussion relevant to the scientific community: How can research on COI disclosure balance the need for transparency with respect for individual privacy? Such debates are essential to guide both future research practices and policy decisions in scientific societies.

**Items reported in the COI form provided by Europerio 11**

Type

Receipt of grants/research supports

Receipt of honoraria or consultation fees

Participation in a company sponsored speaker´s bureau

Stock share holder

Spouse/partner

Other support (please specify)
