# Supplementary material for: Transparency and conflicts of interest disclosure at an international periodontology conference
Source: Eur J Oral Sci. 2026 Mar 12;134(3):e70075. doi: 10.1111/eos.70075 (PMC13181539; doi:10.1111/eos.70075)
Supplement: Supplementary file 2 — Supporting Information [file EOS-134-e70075-s001.pdf]

European
Federation of
Periodontology

Approved 12th April 2024 in Zagreb

EuroPerio PROCEDURES

These Procedures have been prepared by the Congress Committee of the EFP and shall be considered as guidelines for the organisation of EuroPerio Conferences. The Procedures are based particularly on the experiences gained from previous EuroPerio Conferences.

Article 1 - Definition of EuroPerio

EuroPerio is the Scientific Conference of the European Federation of Periodontology (EFP).

EuroPerio is intended for the entire scientific Community engaged in the field of Periodontology and Implant Dentistry, for all Dental/Oral professionals, researchers and students. EuroPerio is held in an EFP full member Society every three years, at a date scheduled by the EFP over a period of at least three days and includes:

- a scientific program
- a commercial exhibition
- a networking programme

In the year of EuroPerio, member National Societies of Periodontology are prohibited from running a national meeting within 3 months either side of EuroPerio.

Article 2 - Venue

Selection of the Venue for the EuroPerio Congress will be made by the Executive Committee in the autumn meeting of year -6 prior to the Congress. For details on procedures for Venue Selection refer to document “EuroPerio-Venue Selection”, which is available on the EFP website and from the EFP Congress Committee Chairman.

Article 3 – Organizing Committee Selection

3.1 Selection of EuroPerio Organising Committee

In the first week of November of year -5, the Congress Committee Chairman and Secretary General will make a call for applications for the position of EuroPerio Chairman for the next EuroPerio. This call for applications will be widely publicised within the EFP community via channels like the EFP Website or electronic mail to all EFP National Member Societies´ current Presidents and EFP delegates.

Interested EuroPerio Chairmen candidates shall send their full candidacy applications to the Secretary General and Congress Committee Chairman by December 15 (year -5) including the proposal by the candidate EuroPerio Chairman for the positions of Scientific Chairman and EuroPerio

Treasurer. The candidacy should include brief CVs, specific details of previous experience of conference organisation for all nominees and all necessary letters of support as per stipulations below.

Proposals for EuroPerio Organising Committee Teams will be reviewed for eligibility by the EFP General Secretary, EFP Treasurer, EFP President and Congress Committee Chairman. For practical reasons, a short list of applications according to defined criteria, such as level of experience in the organization of large scale international meetings.

Valid proposals for EuroPerio Organising Committee Teams will be distributed to full EFP National Member Societies at least 6 weeks prior to the General Assembly in the following year (year -4).

The General Assembly votes for one EuroPerio Organising Committee amongst the candidate teams (year -4).

3.2 Profile EuroPerio Chairman

- Active member of a full EFP National Member Society
- Enthusiastic, pro-active, well-organized person, with strong communication skills, and one who is aware of all tasks and responsibilities related to this position
- Previous experience in organisation of major international meetings and knowledge of the EFP
- Candidacy is officially supported by his/her full EFP National Member Society
- Candidacy is endorsed by one active or retired member of the Executive Committee or previous EuroPerio Organising Committee Member;
- Has not held this position before.

3.3 Profile other Members EuroPerio Organising Committee

- The Scientific Chairman and EuroPerio Treasurer shall come from different EFP National societies, other than the EuroPerio Chairman.
- Previous experience in organisation of meetings and knowledge of the EFP
- Candidacy is officially supported by his/her full EFP National Member Society

The local national society where the EuroPerio venue is located will be asked to nominate one member to act as National Society Representative on the EuroPerio Organising Committee,

The organising committee of EuroPerio will thus constitute these 4 members plus the Congress Committee Chair.

The EFP Secretary General, the EFP Treasurer, the EFP President and one EFP Vice President will be invited *ex-officio* to attend committee meetings of the EuroPerio organising committee.

Article 4 - Organisation

4.1 Official Organiser

The EFP is the official organiser of the Congress and bears the organisational and financial responsibility of the Congress.

4.2 EuroPerio Chairman

The EuroPerio Chairman is the Director of the Organising Committee. The EuroPerio Chairman works closely with the other members of the Organising Committee, the EFP Coordinator and the CorePCO appointed by the EFP.

The EuroPerio Chairman works in accordance with the Congress Committee who reserves the right to intervene in the case of conflict with EFP goals or standards.

To ensure continuity and improve communication, the incoming EuroPerio Chairman will participate, as an observer, in the meetings of the Organising Committee of the previous EuroPerio.

The EFP Head of Operation will be invited to all the meetings.

4.3 Organising Committee

The Organising Committee under the direction of the EuroPerio Chairman includes a Scientific Chairman, an EuroPerio Treasurer, a National Society Representative, and the Congress Committee Chairman.

The EFP Treasurer, the EFP Secretary General, the EFP President and one EFP Vice President are ex-officio members of the Organising Committee. The EFP Treasurer supervises the financial management of the conference (see 5.1) and leads the contact with EFP partners (see 5.2).

4.4 EFP Coordinator

The EFP Coordinator acts as liaison officer to the EFP National Societies. Executive Committee and other EFP Committees.

The EFP Coordinator provides administrative support to the Organising Committee, if requested.

The EFP coordinator manages the logistics (room, technical set up, catering, dinners) for EuroPerio Organising Committee meetings in the framework of the fall EFP Executive Committee meeting and annual General Assembly.

4.5 CorePCO

The CorePCO, nominated by the EFP, manages the Congress organisation on a day-to-day basis in close collaboration with the EuroPerio Chairman and the Organising Committee.

The CorePCO reports to the Congress Committee.

Article 5 – Guidelines for Important Organisational Areas

5.1 Financial management

Financial management of the meeting is ensured by the Organising Committee. The EuroPerio Treasurer follows in detail the evolution of the budget which is developed, monitored and updated by the CorePCO, and keeps the EFP Treasurer informed .

The EuroPerio Treasurer sends to the Congress Committee an initial complete budget 2 years before the Congress. As from this date, she/he sends every quarter an update of the evolution of the financial situation.

An advance of funds is given by the EFP, in accordance with the EFP Treasurer, 3 years before the Congress when requested by the Organising Committee.

All necessary guarantees should be taken, where feasible, in order to minimise financial risk.

The definitive accounts should be closed at the latest 6 months after the Congress. Any incidental surplus of the Conference shall be distributed between EFP Member Societies and EFP according to the formula agreed by the General Assembly.

5.2 Sponsorship

The aim of partnership with the Industry or other commercial Societies is to collect the necessary funds for the EuroPerio organisation, while trying to minimise the attendees' registration fees.

The EuroPerio Treasurer and the Core PCO, in collaboration with the Organising Committee, drafts Sponsorship opportunities with different participation levels according to the possibilities offered by the general organisation and venue.

The EuroPerio Treasurer leads the contacts with potential industry partners in collaboration with the Organising Committee and the CorePCO.

The EFP Treasurer leads the contacts with EFP partners in collaboration with the Organising Committee and the CorePCO.

These contacts can be made up to 3 years before the Congress i.e. following the end of the previous EuroPerio Congress.

5.3 Commercial exhibition

The cost for the space rental/booths is fixed according to the budget requirements and Sponsorship partnership modalities.

Contact with the exhibitors are ensured by the CorePCO in collaboration with the Organising Committee. These contacts can only take place 3 years before the Congress, following the end of the previous EuroPerio Congress.

The list of exhibitors is submitted to the Congress Committee, who control any issues regarding scientific integrity and reserve the right to intervene in the case of conflict.

5.4 Registration fees

The cost of registration fees is approved by the General Assembly of the EFP on the proposal of the Organising Committee, in the calendar year before the Congress.

A scale of registration fees fixes different categories of attendees. Members of National Societies should benefit from reduced registration fees compared with non-members. Full time students who can give proof of their status, and Dental Hygienists will benefit from markedly reduced registration fees.

According to a calendar determined by the Organising Committee, the different categories can benefit from reduced fees for advanced registration.

Current members of the EFP Executive Committee, the EuroPerio Organising Committee, and Past Presidents and Committee Chairs of the EFP will be offered free registration to the Conference. Other cases of free registrations can be offered by the decision of the Organising Committee.

Group registrations may receive reduced fees in accordance with the Congress Committee on the proposal of the Organising Committee.

A limited number of invitations can be offered to the sponsors and exhibitors as long as this number is previously mentioned in the document provided to the partners and exhibitors.

The EuroPerio Chairman reserves the right to have his/her own guests in accordance with the Congress Committee.

5.5 Scientific program

The scientific program of EuroPerio is prepared by the Scientific Chairman in collaboration with the Organising Committee and should be presented to the General Assembly in the year before the Congress.

Before this date, the National Societies of the EFP are invited by the Scientific Chairman to propose possible speakers and topics for the Congress to the Organising Committee in order to help the Organising Committee in finalising a balanced programme, and in introducing if possible new subjects and speakers.

The scientific program includes:

- an Opening Ceremony
- a General Program composed of several Sessions
- several Free Sessions on research and clinical reports
- a Poster Session
- a sponsored program devoted to new developments in Industry composed of several sessions (Industry Sessions)

The schedule and room allocation is fixed by the Organising Committee according to the possibilities offered by the general organisation of the Congress.

Opening Ceremony

The Opening Ceremony should enhance the prestige of EuroPerio. The Opening Ceremony should mark the opening of the Congress, but can be preceded by regular congress sessions and by activities relevant to EuroPerio, such as, satellite meetings, sponsor sessions, and special interest group sessions.

The program of the Opening Ceremony is planned by the Organising Committee in accordance with the Congress Committee. The Opening Ceremony is led by the EuroPerio Chairman and may include the participation of local authorities, EFP Representatives, Organisation Committee members, and an exceptional conference speaker chosen for his international fame in a scientific or cultural field, as well as an artistic/creative event

General Program

The General Program is determined by the Organising Committee on the proposal of the Scientific Chairman in accordance with the Congress Committee.

The Organising Committee can propose a General Theme useful for the promotion of the Congress, but all the current topics should be included in order to make the Congress as attractive as possible. There should be a balance between basic and clinical topics in order to satisfy a wide audience.

The General Program is composed of Sessions that have a similar format and structure, and have a limited number of speakers. For each Session a Chair (Session Chair) has the task to present the theme of the conference, to introduce the other Conference Speakers and to moderate the discussion sessions. The selection of the Speakers and Session Chair are done by the Organising Committee on the proposal of the Scientific Chairman, but no Conference Speakers should be officially contacted before the scientific program is presented to the General Assembly. For equal competencies, European Speakers should be preferred. One or several conferences of the General program are for Dental Hygienists

All the invitations to speakers are issued in the calendar year before the Congress. The definitive General Program should be finalised 14 months before the Congress. Speakers of EuroPerio congress should refrain from presenting/speaking in other EFP National Society meetings 3 months before or after EuroPerio. The invited Session Chairmen and Conference Speakers are exempted from their registration fees and will receive compensation for travel and accommodation, according to modalities fixed by the Organising Committee. The invited Session Chairmen and Session Speakers do not receive honorarium for their participation.

Abstracts

A call for abstracts should be widely broadcasted by the means used for promotion, 1 year before the Congress. Abstracts should be drawn up according to precise instructions determined by the Organising Committee in collaboration with the Chairman of the Selection Committee.

The abstracts are submitted by their authors via an electronic abstract handling system supervised by the Organising Committee before a set deadline date which will be approximately 6 months before the Congress.

Abstract Selection Committee

The Abstract Selection Committee includes a Chairman and a minimum of three individuals from EFP Member Societies. They will recruit additional abstract reviewers as required. The Scientific Chairman should participate in this process either as Committee Chair or in his own right.

The Selection Committee is nominated by the Organising Committee. It is in charge of reviewing all the abstracts sent to the Organising Committee, and selecting those to be accepted to the meeting. In addition the committee will select the mode of presentation for each accepted abstract and will organise them into appropriate clinical and scientific themes.

If the rating and conclusion of abstracts cannot be completed via an electronic abstract handling system, the Selection Committee meets 5 months before the Congress and transmits its conclusions to the Organising Committee at the end of this meeting. The EuroPerio Chairman, the Scientific Chairman, and the EuroPerio Treasurer may take part in the meeting of the Selection Committee.

The Members of the Selection Committee, are exempted from paying registration fees to the meeting. and receive compensation for travel and accommodation, according to modalities fixed by the Organising Committee. ***Free Oral Sessions***

Free oral sessions devoted to research and clinical reports are chosen by the Selection Committee and organised in appropriate themes.

Each free session is directed by a Session Chair nominated by the Organising Committee. Session Chairs are exempt from registration fees but their travel and accommodation are not paid by EuroPerio.

Speakers participating in the free sessions are not exempt from registration fees, and their travel and accommodation are not paid by EuroPerio.

Competitions awarded by a prize offered by a sponsor or an institution can be organised. All the abstracts selected for communication will be published in a special issue of the Journal of Clinical Periodontology in accordance with a contract signed with the Editor. Speakers invited to present in a competition (Jaccard Prize) are excempt from registration fees.

Poster Session

Poster sessions are organised by the Organising Committee according to the same modalities as those of the free sessions. A standard format for the Posters is determined by the Organising Committee in collaboration with the Chairman of the Selection Committee. The Poster participants are not exonerated from registration fee and their travel and accommodation are not paid by the EFP.

Industry Sessions

Industry sessions are part of the presentations offered within the framework of the partnership.

The subjects, Speakers and Chairmen are proposed to the Organising Committee by the Industry partner and all the proposals are submitted to the Congress Committee who control the scientific integrity and reserve the right to intervene in case of conflict.

Costs for the Speakers and Chairmen participating in the Industry sessions are covered partly by the Industry partner, according to their personal agreement.

5.6 Translations

English is the official language of the EFP and EuroPerio. All sessions and free communications should be in English.

EuroPerio can provide simultaneous translation for main sessions into any other language on the request of a National Society, which takes responsibility of costs.

5.7 Networking Programme

The aim of the Networking Programme organised within the framework of EuroPerio is to facilitate social exchanges among the attendees in a relaxing manner.

The Networking Programme should enhance the prestige of EuroPerio.

and may include functions such as:

- welcome reception to follow the opening ceremony
- a Speakers' Dinner offered to invited faculty as well as special guests invited by the EuroPerio Chairman.
- a congress party (?)
- An Official Congress Evening opened to all the participants with the smallest possible financial contribution.

The Networking Programme can (partially) be sponsored by Industry Partners or an institution in accordance with the Organising Committee.

National Societies can organise in collaboration with the Organising Committee social events for their members.

Industry Partners can organise social events during EuroPerio for selected guests with the permission of the Organising Committee.

5.8 Promotion and printed matters

The promotional campaign is managed by the CorePCO in collaboration with the Organising Committee. The promotional Campaign starts 3 years before the Congress, at the occasion of the previous EuroPerio Congress.

The promotional Campaign utilises a range of appropriate media and may include:

- Posters widely sent out to scientific Societies world-wide and to the European dental institutions.
- Use of the Journal of Clinical Periodontology in accordance with a contract signed with the Editor.
- Pages of advertisement in other Journals
- Use of Web-related social media platforms
- Direct mailing of flyers, pre-programs, programs and registration forms to all the members of the EFP.
- Mailings of specific documents through EFP National Societies
- Distribution and mailings of specific documents through EuroPerio Industry partners.

National Societies pledge to participate in the promotion to their members by using all possible means. The Organising Committee ensures the printing of all materials that are necessary for the promotion but the National Societies pledge to broadcast these materials to their members. All printed material published for EuroPerio is created by the CorePCO in collaboration with the Organising Committee.
